# Identification of the Bovine Arachnomelia Mutation by Massively Parallel Sequencing Implicates Sulfite Oxidase (SUOX) in Bone Development

**DOI:** 10.1371/journal.pgen.1001079

**Published:** 2010-08-26

**Authors:** Cord Drögemüller, Jens Tetens, Snaevar Sigurdsson, Arcangelo Gentile, Stefania Testoni, Kerstin Lindblad-Toh, Tosso Leeb

**Affiliations:** 1 Institute of Genetics, Vetsuisse Faculty, University of Bern, Berne, Switzerland; 2 Institute for Animal Breeding and Husbandry, Christian-Albrechts-University Kiel, Kiel, Germany; 3 Broad Institute of Harvard and Massachusetts Institute of Technology, Cambridge, Massachusetts, United States of America; 4 Department of Medical Biochemistry and Microbiology, Uppsala University, Uppsala, Sweden; 5 Veterinary Clinical Department, University of Bologna, Bologna, Italy; 6 Department of Veterinary Clinical Sciences, University of Padua, Padua, Italy; University of Liège, Belgium

## Abstract

Arachnomelia is a monogenic recessive defect of skeletal development in cattle. The causative mutation was previously mapped to a ∼7 Mb interval on chromosome 5. Here we show that array-based sequence capture and massively parallel sequencing technology, combined with the typical family structure in livestock populations, facilitates the identification of the causative mutation. We re-sequenced the entire critical interval in a healthy partially inbred cow carrying one copy of the critical chromosome segment in its ancestral state and one copy of the same segment with the arachnomelia mutation, and we detected a single heterozygous position. The genetic makeup of several partially inbred cattle provides extremely strong support for the causality of this mutation. The mutation represents a single base insertion leading to a premature stop codon in the coding sequence of the *SUOX* gene and is perfectly associated with the arachnomelia phenotype. Our findings suggest an important role for sulfite oxidase in bone development.

## Author Summary

Arachnomelia is a defect in skeletal development of cattle. Affected calves are born dead with elongated limbs and facial deformities. The causative mutation for this recessive condition had previously been mapped to a ∼7 Mb interval. We exploited the special structure of cattle families to identify the causative mutation by a purely genetic approach. The rich pedigree records in cattle breeding allowed us to identify the founder animal of arachnomelia, a Brown Swiss bull born in 1957. A few generations later several cattle received two copies of the same chromosome segment from the father of this bull due to inbreeding. One copy was passed through the founder animal and acquired the causative mutation, while the other copy was transmitted through a different line of animals and stayed in its ancestral state. Using next-generation sequencing, we sequenced the entire critical interval in one of these inbred animals. As expected, we found only one single heterozygous position, which consequently represents the causative mutation for arachnomelia. The mutation affects the gene for sulfite oxidase, thus indicating a previously unrecognized important role for this enzyme in bone development. Our findings can immediately be applied to remove this deleterious mutation from the cattle breeding population.

## Introduction

**Figure 1:**
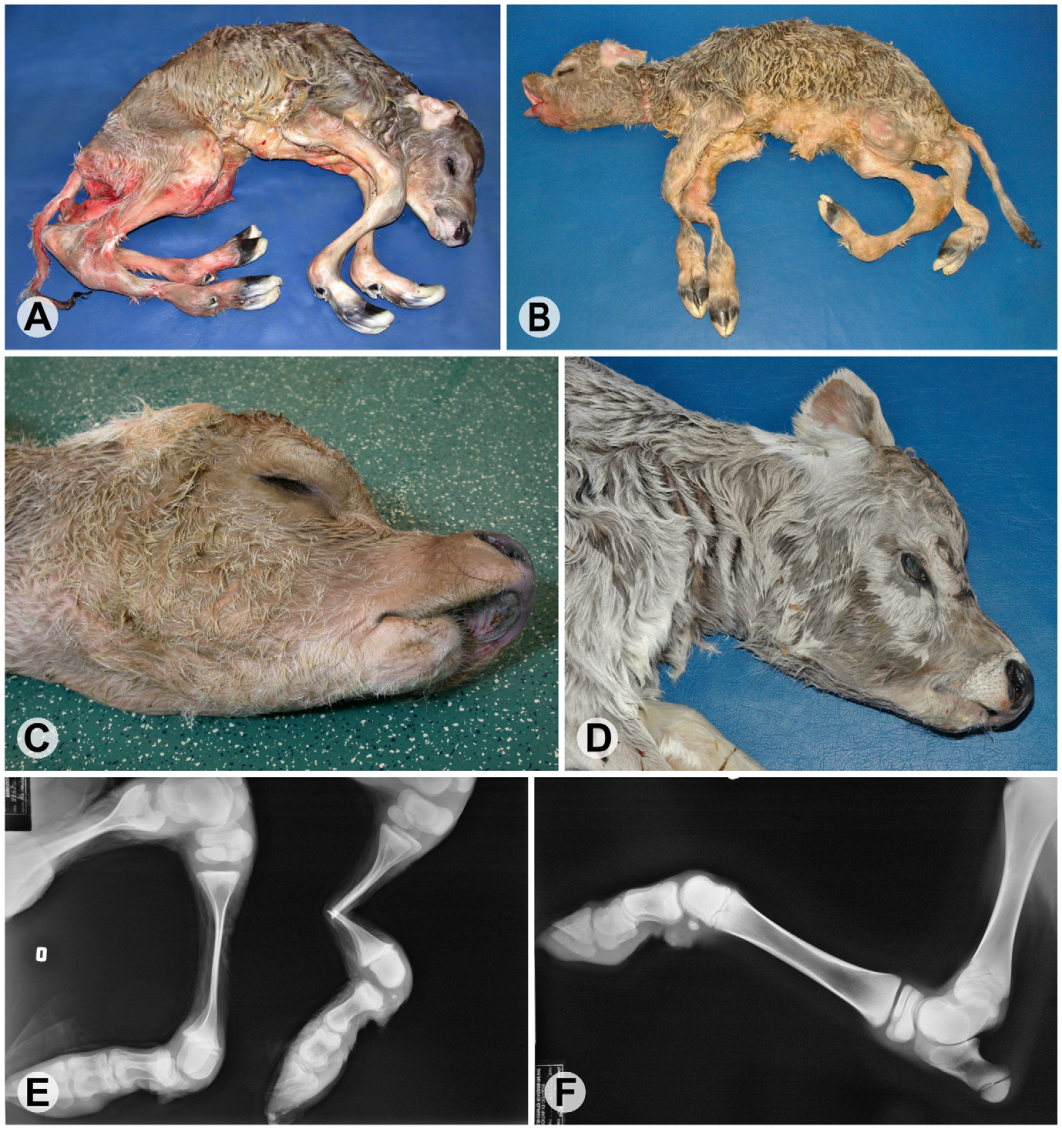
Phenotype of bovine arachnomelia in Brown Swiss cattle. (A,B) Stillborn affected calves; note the abnormal length of all legs (dolichostenomelia), the angular deformities in the distal part of the hind legs with arthrogryposis of the distal joints, and a muscular atrophy of the legs. (C,D) Typical facial deformities of affected calves; note a concave rounding of the dorsal profile of the maxilla and brachygnathia inferior. (E) Radiography of the hind limbs of the affected calf from (C); note that the joint ends (epiphyses) of the long bones are of normal size but with marked thinning of the shafts (diaphyses) showing increased fragility. (F) Radiography of the left hind leg of a non-affected control calf.

Arachnomelia is a genetic disease in cattle characterized by skeletal abnormalities. Affected calves are usually stillborn with a spidery appearance and an abnormally shaped skull (Figure 1). The bones of the limbs are prolonged (dolichostenomelia) with marked thinning of the diaphyses that fracture easily in the course of forced birth assistance. Additional dysmorphic features are variable, e.g. defects of the vertebral column and sometimes cardiac malformations [1]–[4]. Initially, it was assumed that the pathogenesis of bovine arachnomelia resembles that of human Marfan syndrome, which is caused by mutations in the *FBN1* gene [1]. However, the identification of other cattle with a mutation in the *FBN1* gene established that arachnomelia is phenotypically distinct from Marfan syndrome [5]. Arachnomelia affected calves lack some typical Marfan features such as joint laxity and aortic root dilation [3]. Bovine arachnomelia is inherited as a monogenic autosomal recessive trait with complete penetrance [2], [4]. Carrier animals do not present any clinical signs. An outbreak of arachnomelia with hundreds of cases occurred during the 1980s after extensive world-wide usage of a highly selected artificial insemination sire in the international Brown Swiss cattle population [2]. In the past few years, a comparable outbreak of arachnomelia occurred in German Fleckvieh cattle [4]. Different genes seem to be responsible for the arachnomelia disease in these two cattle breeds, as there is no relationship between the founder animals and recently two independent loci were genetically mapped to different chromosomes [6], [7]. We mapped the arachnomelia mutation in Brown Swiss cattle to a 7.19 Mb interval on bovine chromosome (BTA) 5 [6]. Due to the lack of suitable candidates the genetic basis of arachnomelia is not understood. Therefore, the spontaneous cattle arachnomelia mutants provide unique resources to gain further insights into the biology of bone development.

Array enrichment and next-generation sequencing technology can be used to rapidly sequence targeted subsets of the genome [8], [9]. Sequence capture enrichment has already successfully been used to sequence the coding portion of the human genome [10]–[12] and also large genomic intervals [13], [14]. This technology offers great potential for positional cloning projects where the mapping resolution may be limited, e.g. in the case of recent mutations. Thus megabase sized regions can now be re-sequenced efficiently. Furthermore, the unique family structures in livestock populations greatly facilitate the discrimination of the causative variant from the many neutral polymorphisms that have to be expected from such re-sequencing projects. In this report we applied this approach to identify the causative mutation for bovine arachnomelia.

## Results/Discussion

**Figure 2:**
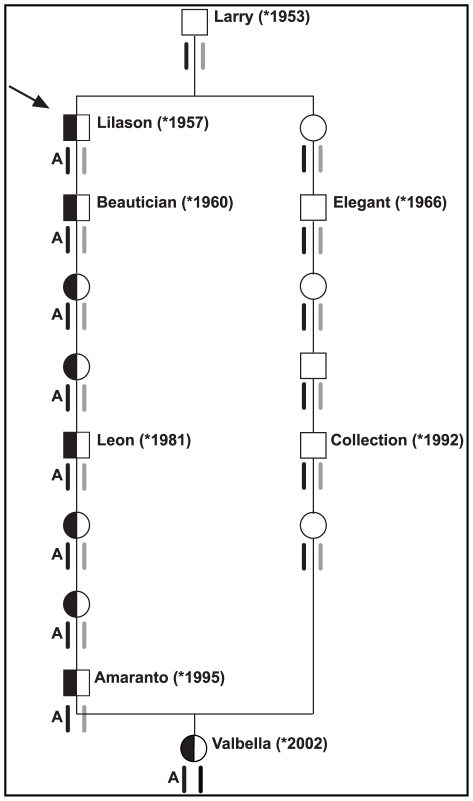
Pedigree of selected inbred Brown Swiss cattle. The special structure of livestock families facilitates the mutation identification: The bull Lilason (arrow) is the founder animal for arachnomelia. Thus the arachnomelia mutation (indicated by an A) must have occurred either in the germline of Larry or during the early embryonic development of Lilason. Beneath the animals the two copies of BTA 5 are indicated. Black symbols represent the ancestral chromosome, on which the mutation occurred. Gray symbols represent any other wild-type copy of BTA 5. Due to the inbreeding loop, the cow Valbella has inherited two copies of the same chromosome from her ancestor Larry. Her paternal copy of the critical 7.19 Mb interval carries the arachnomelia mutation, while her maternal copy is still in its ancestral wild-type state. The BTA 5 haplotypes were confirmed by microsatellite genotyping of available samples from Beautician, Leon, Amaranto, Elegant, Collection, and Valbella [5].

We selected two individuals for an array-based sequence capture and massively parallel sequencing approach to re-sequence the entire critical interval. Our design included one arachnomelia affected calf assumed to be homozygous across the entire sequence interval including the causative variant. The other animal chosen for re-sequencing was a partially inbred non-affected cow. Based on pedigree and marker data this non-affected cow was identical-by-descent for the critical segment of BTA 5, except for the causative arachnomelia mutation, which we predicted to reside only on her paternally derived chromosome (Figure 2). We chose this cow for complete re-sequencing of the entire critical interval, as the detection of a heterozygous polymorphism in this animal should reveal the causative mutation.

We enriched the ∼3.5 Mb non-repetitive sequence within the critical interval of BTA 5 in the two animals and collected about 30 million illumina reads per animal. After the alignment to the reference sequence, the mean coverage was 44-fold for the affected animal and 15-fold for the non-affected animal. The difference was most likely due to technical variations during the hybridization. The depth of coverage was variable across the targeted interval, similar to what had been described previously [15]. However, the two animals showed a similar distribution of the gaps across the targeted interval (Figure S1). In the affected calf 96% of the enriched bases had at least 4-fold coverage compared to 91% in the control sample. We called a homozygous variant when the respective position had ≥4-fold coverage and the observed difference between the experimental reads and the reference sequence occurred at ≥75% frequency. For the calling of a heterozygote variant we applied a threshold of ≥15-fold sequence coverage and a variant allele frequency between 25% and 75% based on published recommendations [16]. Using these criteria we recovered a total of 6,025 putative variants between the reads and the reference for the affected calf (4,848 homozygous and 1,177 heterozygous), and 4,318 putative variants for the control cow (3,818 homozygous and 500 heterozygous, Table 1). The high number of seemingly heterozygous variants detected in two animals supposed to be completely or almost completely homozygous underscores the challenges of aligning short reads from a complex mammalian genome to a draft quality reference sequence. About three-fourths of the heterozygous variant calls were located within an olfactory receptor (*OR*) gene cluster encompassing a 2.2 Mb segment with a total of 136 annotated loci [17]. The read coverage within this region was significantly higher than the average (60/37-fold) indicating segmental duplications. Other factors contributing to the high number of putative heterozygous variants may have been sequencing errors, non-specific hybridization of DNA during the enrichment, or amplification-mediated artifacts (i.e. polymerase errors during library preparation).

**Table 1:** Sequence variants identified by massively parallel sequencing.

	Case	Control	Shared
Total variants	6,025	4,318	3,083
SNPs	5,151	3,827	2,841
Transitions	3,255	2,544	1,903
Transversions	1,896	1,283	938
InDels	874	491	242
Insertions	477	241	95
Deletions	397	250	147
Homozygous (variant freq. ≥75%; coverage ≥4)	4,848	3,818	2,972
Heterozygous (25%< variant freq. <75%; coverage ≥15)	1,177^a^	500^a^	111
Coding variants			
Homozygous CDS/splice site (coverage ≥4)	79	63	76
Homozygous CDS/splice site (coverage 1-3)		5	
Missing coverage CDS/splice site		8^b^	
Heterozygous CDS/splice site (coverage ≥10)		3^c^	

**^a^**Most of these heterozygous positions (case: 74.4%, control: 61.2%) are located in the region of the *OR* gene cluster, which represents only 31% of the target sequence.

**^b^**Sanger re-sequencing revealed that these eight positions were all homozygous mutant.

**^c^**Sanger re-sequencing revealed that only one of these three positions was a true heterozygous variant.

**Figure 3:**
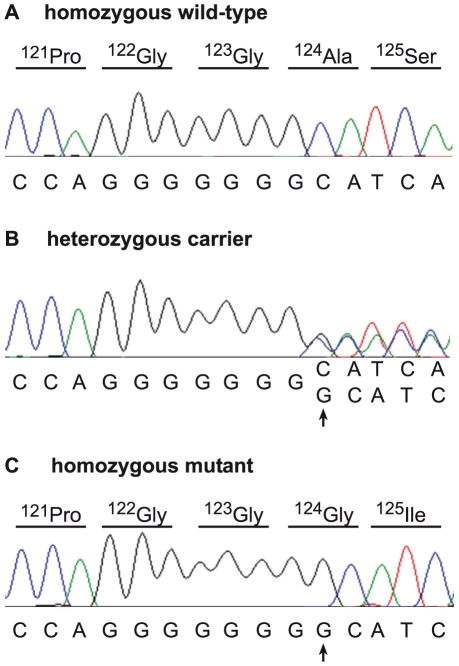
Mutation identification in the bovine *SUOX* gene. Electropherograms of a wild-type control animal (A), a carrier cow (B), and an arachnomelia affected calf (C) are shown. A single G insertion, which was homozygous in all analyzed cases, is marked by an arrow. The protein translation of the wild-type and mutant sequence is shown above the electropherograms.

Due to the recessive inheritance and the lethal effect of the mutation we hypothesized that most likely a loss of function mutation affecting the coding sequence of a gene would be responsible for arachnomelia. Therefore, we subsequently concentrated on variants that were located within the coding sequences or within the splice sites of the annotated genes in the targeted region of the bovine genome. A total of 79 predicted homozygous variants were located within coding sequences or adjacent splice sites of the affected calf (Table 1, Table S1). The comparison between the affected calf and the non-affected cow revealed that 68 of these variants had identical homozygous mutant genotypes in the control cow and could thus be excluded as causative variants. Eight variants had no illumina coverage in the control cow and were subsequently found to be homozygous mutant by Sanger sequencing and thus also excluded. The three remaining variants were putative heterozygous variants in the control cow with 10, 12, and 27-fold coverage, respectively. We validated these three potential variants by Sanger sequencing and found that two of them were false positives as the affected calf and the control cow shared identical homozygous genotypes for these variants. For the remaining variant we confirmed that the control cow was indeed heterozygous, whereas the affected calf was homozygous mutant compared to the reference sequence. This variant was a 1 bp insertion located in exon 4 of the bovine *SUOX* gene (c.363–364insG, Figure 3). We found perfect concordance between the presence of this insertion and the arachnomelia phenotype (Table 2). All 16 affected calves were homozygous mutant and all 11 available mothers for these animals were heterozygous. Genomic DNA samples of 25 artificial insemination carrier sires, which had recorded arachnomelia offspring and which were related to the assumed founder, were tested and 23 of them were also heterozygous. For each of the two remaining suspected carriers only one single arachnomelia suspicious calf was recorded by the breeding organization and the diagnoses of these calves had not been confirmed by veterinarians. Therefore, we think that these two reported arachnomelia calves represented phenocopies and their sires are indeed free of the arachnomelia mutation. Our material included Beautician, son of the assumed founder Lilason, who was responsible for spreading the mutation into the global Brown Swiss population. We confirmed that Beautician is heterozygous for the *SUOX* mutation supporting the hypothesis for the origin of the causative mutation. Three acknowledged carrier bulls, which had the same genetic constellation as the non-affected inbred cow of our mutation analysis and were homozygous for all tested markers in the critical interval, were also found to be heterozygous for the *SUOX* mutation (Figure S2). None of 309 unrelated healthy Brown Swiss cattle had the homozygous mutant genotype, but 10 of them were presumed carriers. Thus the allele frequency of the deleterious insertion within this sample of unrelated Brown Swiss cattle was 1.6%, which is about half of the frequency that was estimated 20 years ago [18]. We screened a genetically diverse panel of animals from 15 breeds widely used in commercial cattle production to confirm that the identified mutation does not occur outside the Brown Swiss population. None of the 150 chromosomes in this sample showed the causative insertion (Table 2).

**Table 2:** Association of the 1 bp insertion with the arachnomelia phenotype.

	wt/wt	wt/ins	ins/ins
Arachnomelia affected calves	-	-	16
Carriers^a^	2^b^	34	-
Unrelated healthy Brown Swiss cattle	299	10	-
Healthy control cattle from 15 other breeds	75	-	-
Total	376	44	16

**^a^**Parents of affected offspring were classified as carriers.

**^b^**The affected offspring of these two animals were assumed to represent phenocopies (see main text).

The c.363–364insG insertion is predicted to result in a frameshift beginning with amino acid residue 124 in the bovine SUOX protein sequence (p.Ala124GlyfsX42). While it is unclear whether the mutant protein of 164 residues is actually expressed, with more than 75% of the normal SUOX protein missing, it is very unlikely that the mutant protein fulfills any physiological function. Due to the frameshift and the premature stop codon, any mutant protein produced will contain 42 altered amino acids, and could potentially interfere with normal cellular function. The *SUOX* gene encodes the molybdohemoprotein sulfite oxidase, a terminal enzyme in the oxidative degradation pathway of sulfur-containing amino acids. Each monomer of the dimeric SUOX enzyme consists of three domains, the N-terminal heme domain, the central molybdenum domain and a C-terminal domain [19]. Deficiency of this enzyme in humans usually leads to recessive inherited sulfocysteinuria (OMIM 272300) characterized by major neurological abnormalities and early death [19]–[22]. The more severe cases, characterized by frequent seizures and death within a few days of birth, result from a complete SUOX loss of function [20], [22]. A single case of human sulfocysteinuria caused by a 1 bp deletion of human *SUOX* leading to a truncated protein has been reported, where some dysmorphic skeletal features were diagnosed in addition to severe neurodevelopmental anomalies [22].

The arachnomelia phenotype in cattle shows more severe skeletal defects and neonatal lethality compared to the human sulfocysteinuria patients. Another genetic disorder of human sulfur metabolism associated with a bone phenotype (arachnodactyly) is caused by *CBS* mutations resulting in cystathionine beta-synthase deficiency [23].

In cattle there are two virtually identical arachnomelia phenotypes in Brown Swiss cattle and in German Fleckvieh cattle. The mutation in German Fleckvieh was mapped to BTA 23, a region where the *MOCS1* gene encoding molybdenum cofactor synthesis 1 is located [7]. A candidate causative mutation for German Fleckvieh arachnomelia has recently been identified in the bovine *MOCS1* gene [Johannes Buitkamp, personal communication]. The MOCS1 protein is required for the synthesis of the molybdopterin cofactor, which forms the active site in SUOX. The involvement of SUOX and MOCS1 in the same biochemical pathway mutually supports the causality of these mutations in Brown Swiss and German Fleckvieh cattle.

We think that we have established the causality of the *SUOX* mutation for arachnomelia in Brown Swiss cattle based on the following arguments: (1) the perfect association of the *SUOX* mutation to the arachnomelia phenotype, (2) the obvious functional impact of a frameshift mutation on the encoded protein, (3) four non-affected inbred animals, which were identical-by-descent for all tested markers across the critical interval, were heterozygous at the *SUOX* mutation. The recognition of these four inbred animals was a key element in our discovery and illustrates the potential of livestock specific population structures for genetic research. This study represents one of the first successful applications of microarray-based enrichment of megabase-sized genomic regions followed by massively parallel sequencing to unravel the causative mutation underlying a Mendelian trait. This technology significantly reduces the time and resources required for mutation identification by abrogating the need for high resolution genetic mapping and thousands of Sanger sequencing reactions.

In summary, we have successfully applied a sequence capture strategy to identify *SUOX* as the causative gene for bovine arachnomelia, and thereby discovered an essential role for this gene during bone development. The knowledge of the causative mutation will allow direct genetic testing of Brown Swiss cattle and the elimination of this fatal genetic disease from the breeding population. This study highlights the enormous potential of spontaneous mutants in domestic animals to gain further insights into mammalian biology.

## Materials and Methods

### Sequence capture enrichment

The bovine genome assembly Btau 4.0 was used for all analyses. A custom tiling 385k sequence capture array targeting the arachnomelia region (BTA 5, 57,285,788–64,478,535 bp) was designed and manufactured by Roche NimbleGen. The reference sequence contained 179 gaps with a total of 49,558 bp (0.7% of the target sequence). The array was designed using NimbleGen's standard 15-mer frequency masking to minimize repeat content within capture probes. The probe spacing, tiling overlap, and probe length were determined using proprietary algorithms (NimbleGen). For the sequence capture library construction a total of 20 µg high-molecular weight genomic DNA was sheared to yield approximately 400 bp fragments using an ultrasound device and purified on QIAquick columns (QIAGEN). The genomic DNA was polished and repaired using T4 DNA polymerase and T4 PNK (Fermentas). Illumina adapters were added to each genomic DNA sample using T4 DNA ligase (Fermentas). Adapter ligated samples were purified and amplification competency was assessed by PCR with primers complementary to the ligated adapters and finally evaluated by agarose gel electrophoresis. Array hybridization was executed using an X1 mixer (Roche NimbleGen) and the NimbleGen Hybridization System for 3 days at 42°C following the manufacturer's recommended conditions. Human Cot-1 DNA (Invitrogen/Life Technologies) was used at a mass ratio of 5:1 vs. the library. Arrays were washed using the recommended protocol (Roche NimbleGen Arrays User's Guide v2.0). The captured molecules were eluted from the slides with elution reagent using a NimbleGen Elution Station. Eluted molecules were dried by centrifugation under vacuum, rehydrated and PCR amplified for 18 cycles with Phusion polymerase (Finnzymes). Enrichment of samples was assessed by quantitative PCR comparison to the same samples prior to hybridization. Following evaluation by agarose electrophoresis and purification, the amplified capture libraries were processed into sequencing libraries for the illumina GAII. A total of 33,676,855/31,356,905 single reads of different length (36 or 76 bp) comprising 2,245,122,900/2,241,558,140 bases raw data were produced for case and control, respectively.

### Read mapping and variant calling

Repetitive elements of the 7.19 Mb genome sequence were masked using the Repeatmasker software and a total of 3,542,013 bp single copy sequence was used as reference for short read mapping. The SeqMan NGen v2.0 software (DNASTAR) was used for assembly with a minimal match percentage of 97%, a minimal match size of 19 nt, and a maximal coverage of 200. A total number of 4,984,147/1,440,459 reads were assembled for the case and the control, respectively. We required a minimal coverage of 4-fold for variant detection and ≥75% of the variant allele for calling a homozygous mutant genotype. Variants falling within the first 10 nt adjacent of masked repetitive sequences were excluded due to obvious alignment inconsistencies (e.g. high coverage, probably due to incomplete masking at the ends of repeats). The post-assembly processing of the variant data was carried out using the R statistical software package [24].

### Sanger re-sequencing

Some variants were genotyped by re-sequencing of targeted PCR products using Sanger sequencing technology. PCR products were amplified using AmpliTaqGold360Mastermix (Applied Biosystems). PCR products were directly sequenced on an ABI 3730 capillary sequencer (Applied Biosystems) after treatment with exonuclease I and shrimp alkaline phosphatase. Sequence data were analyzed with Sequencher 4.9 (GeneCodes).

## Supporting Information

Figure S1 Capture performance. In both samples >90% of the bases in the capture target region had at least 4-fold coverage (96% in the affected calf and 91% in the control). The non-captured single copy sequence regions are shown as the proportion of bases (in percent) per sliding 10 kb window. The distribution of the capture gaps was very similar between our two samples. More than 80% of gaps without sequence reads occurred in similar regions of single copy sequence.
(0.03 MB PDF)

Figure S2 Pedigree of selected Brown Swiss cattle. The bull Lilason (arrow) is the acknowledged founder animal for arachnomelia. His son Beautician was extensively used as artificial insemination sire and spread the deleterious mutation into the international Brown Swiss population. Chromosome symbols beneath the animals indicate that we analyzed their BTA 5 haplotypes by microsatellite markers. The ancestral BTA 5 haplotype, on which the arachnomelia mutation occurred, is indicated in solid black. Any other BTA 5 haplotype is indicated in gray. The arachnomelia mutation is denoted by the letter A. We identified four inbred animals (underlined), which inherited both of their BTA 5 haplotypes in the critical region from their ancestor Larry. These animals were homozygous for all tested markers across the critical interval with the exception of the *SUOX* c.363-364insG mutation.
(0.03 MB PDF)

Table S1 Details on the 79 coding polymorphisms.
(0.04 MB XLS)
