# Supplementary material for: Identification of the Bovine Arachnomelia Mutation by Massively Parallel Sequencing Implicates Sulfite Oxidase (SUOX) in Bone Development
Source: PLoS Genet. 2010 Aug 26;6(8):e1001079. doi: 10.1371/journal.pgen.1001079 (PMC2928811; doi:10.1371/journal.pgen.1001079)

Figure S1. Capture performance. In both samples > 90% of the bases in the capture target region had at least 4-fold coverage (96% in the affected calf and 91% in the control). The non-captured single copy sequence regions are shown as the proportion of bases (in percent) per sliding 10 kb window. The distribution of the capture gaps was very similar between our two samples. More than 80% of gaps without sequence reads occurred in similar regions of single copy sequence.

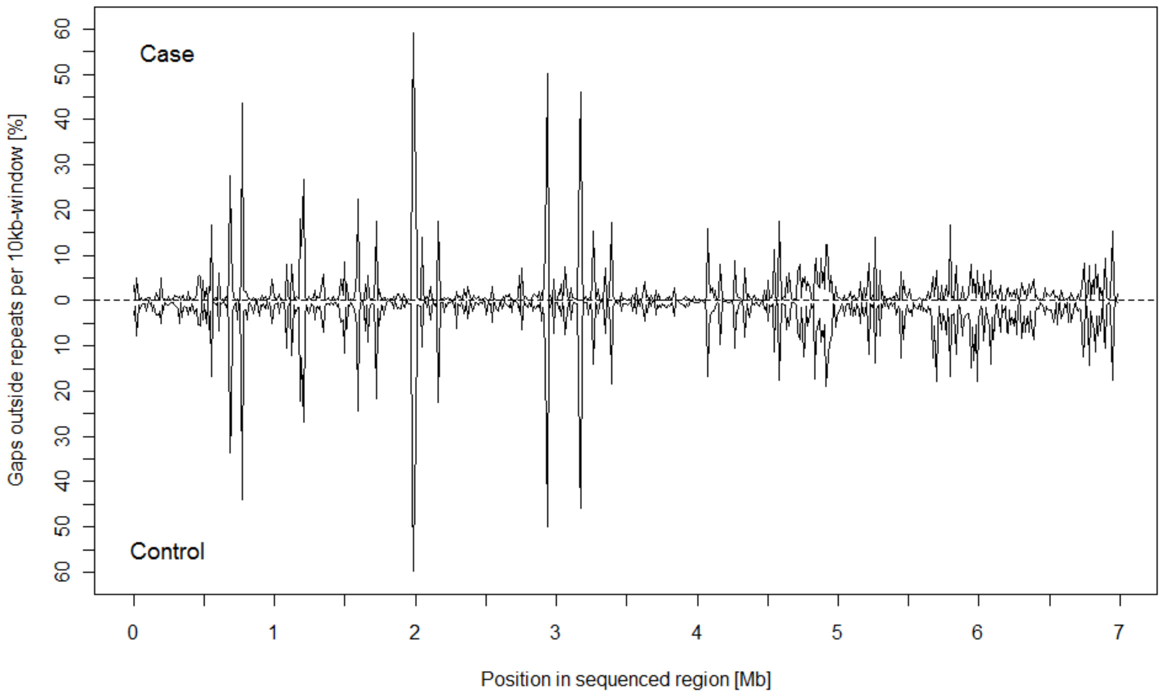

Supplement: Figure S1 — Capture performance. In both samples >90% of the bases in the capture target region had at least 4-fold coverage (96% in the affected calf and 91% in the control). The non-captured single copy sequence regions are shown as the proportion of bases (in percent) per sliding 10 kb window. The distribution of the capture gaps was very similar between our two samples. More than 80% of gaps without sequence reads occurred in similar regions of single copy sequence. (0.03 MB PDF) [file pgen.1001079.s001.pdf]
