# Supplementary material for: Identification of the Bovine Arachnomelia Mutation by Massively Parallel Sequencing Implicates Sulfite Oxidase (SUOX) in Bone Development
Source: PLoS Genet. 2010 Aug 26;6(8):e1001079. doi: 10.1371/journal.pgen.1001079 (PMC2928811; doi:10.1371/journal.pgen.1001079)

Figure S2. Pedigree of selected Brown Swiss cattle. The bull Lilason (arrow) is the acknowledged founder animal for arachnomelia. His son Beautician was extensively used as artificial insemination sire and spread the deleterious mutation into the international Brown Swiss population. Chromosome symbols beneath the animals indicate that we analyzed their BTA 5 haplotypes by microsatellite markers. The ancestral BTA 5 haplotype, on which the arachnomelia mutation occurred, is indicated in solid black. Any other BTA 5 haplotype is indicated in gray. The arachnomelia mutation is denoted by the letter A. We identified four inbred animals (underlined), which inherited both of their BTA 5 haplotypes in the critical region from their ancestor Larry. These animals were homozygous for all tested markers across the critical interval with the exception of the *SUOX* c.363-364insG mutation.

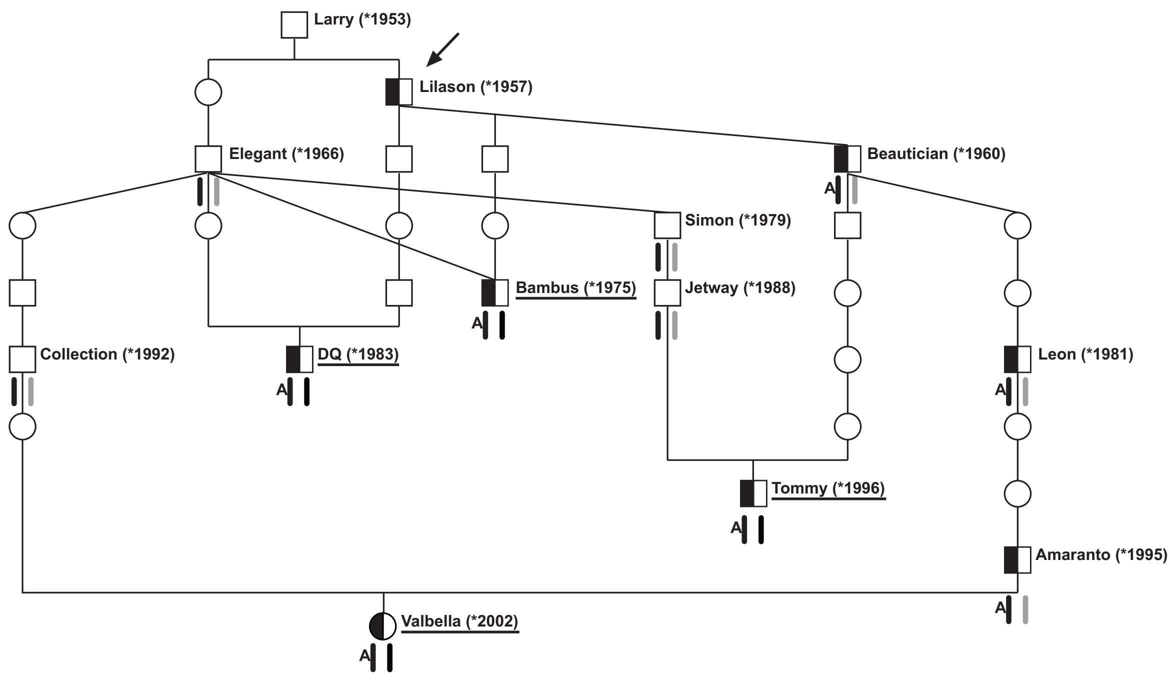

Supplement: Figure S2 — Pedigree of selected Brown Swiss cattle. The bull Lilason (arrow) is the acknowledged founder animal for arachnomelia. His son Beautician was extensively used as artificial insemination sire and spread the deleterious mutation into the international Brown Swiss population. Chromosome symbols beneath the animals indicate that we analyzed their BTA 5 haplotypes by microsatellite markers. The ancestral BTA 5 haplotype, on which the arachnomelia mutation occurred, is indicated in solid black. Any other BTA 5 haplotype is indicated in gray. The arachnomelia mutation is denoted by the letter A. We identified four inbred animals (underlined), which inherited both of their BTA 5 haplotypes in the critical region from their ancestor Larry. These animals were homozygous for all tested markers across the critical interval with the exception of the SUOX c.363-364insG mutation. (0.03 MB PDF) [file pgen.1001079.s002.pdf]
